# Modeling the Perception of Audiovisual Distance: Bayesian Causal Inference and Other Models

**DOI:** 10.1371/journal.pone.0165391

**Published:** 2016-12-13

**Authors:** Catarina Mendonça, Pietro Mandelli, Ville Pulkki

**Affiliations:** 1 Department of Signal Processing and Acoustics, Aalto University, Espoo, Finland; 2 School of Industrial and Information Engineering, Polytechnic University of Milan, Milan, Italy; Kyoto University, JAPAN

## Abstract

Studies of audiovisual perception of distance are rare. Here, visual and auditory cue interactions in distance are tested against several multisensory models, including a modified causal inference model. In this causal inference model predictions of estimate distributions are included. In our study, the audiovisual perception of distance was overall better explained by Bayesian causal inference than by other traditional models, such as sensory dominance and mandatory integration, and no interaction. Causal inference resolved with probability matching yielded the best fit to the data. Finally, we propose that sensory weights can also be estimated from causal inference. The analysis of the sensory weights allows us to obtain windows within which there is an interaction between the audiovisual stimuli. We find that the visual stimulus always contributes by more than 80% to the perception of visual distance. The visual stimulus also contributes by more than 50% to the perception of auditory distance, but only within a mobile window of interaction, which ranges from 1 to 4 m.

## Introduction

Crossmodal interactions are often analyzed in light of the available multisensory perception theories of the time. For most of the twentieth century, these interactions were described by identifying which cue determined the multisensory percept. The terms sensory dominance, capture and ventriloquism were often used [1–6]. In the early 2000s the paradigm shifted away from the winner-takes-all perspective into a more probabilistic approach. Sensory interactions were expected to include weighing processes where the most reliable sensory cue contributed the most to the multisensory percept [7]. The Maximum Likelihood Estimation (MLE) model in particular, which assumes that this weighing process is statistically optimal, has been broadly tested and applied to a number of cue combination cases [8–14].

**Fig 1:**
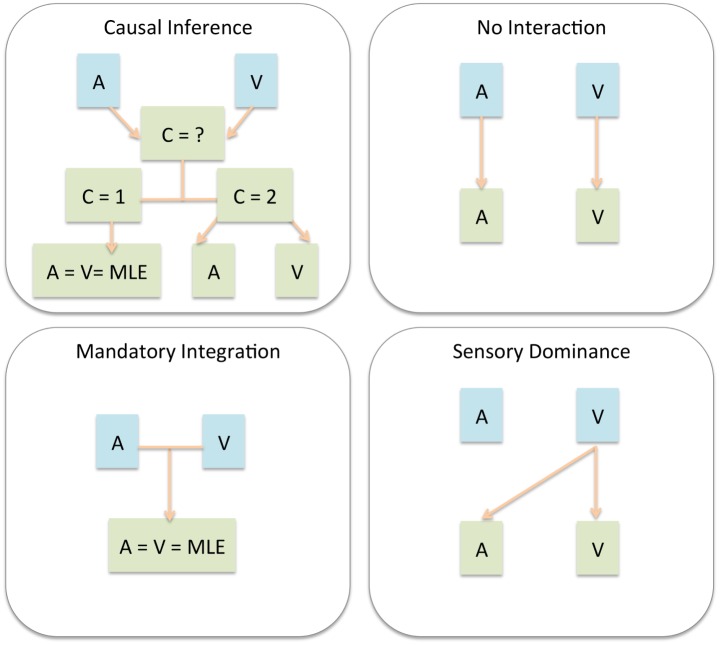
Representation of four models of multisensory interaction. Blue areas represent external events and green areas represent internal events. In *Causal Inference*, the first step is to find the likelihood of the stimuli having been caused by one or two sources. If one source is inferred, then *Mandatory Integration* takes place, which can be predicted by the MLE model. If two causes are inferred, then the estimates will be given by the *No Interaction* model. In the *No Interaction* model each sensory estimate in the multimodal condition can be approximated by the corresponding unisensory percept. In the *Sensory Dominance* model each sensory estimate in the multimodal condition can be approximated by the best unisensory percept. The models are described in detail in the section Multisensory Modeling.

In recent years there has been a significant change in how multisensory interactions are described (see Fig 1 for an illustration of the multisensory models). No longer a unitary percept is expected to arise from multisensory stimulation [15]. It has been proposed that Bayesian causal inference mechanisms can explain human multisensory perception [16–20]. From bisensory stimulation perceivers can infer either one single causal event or two. The higher the temporal or spatial discrepancy, the more likely one is to infer two underlying events. It follows that cue integration is only expected to occur when both stimuli are perceived as stemming from the same physical event. These mechanisms have been shown to describe well the perception of audiovisual horizontal space [19, 20]. However, so far this model was only tested with generative models and using several free parameters. By free parameters we mean unknown values that the model does not predict. As a way of calculating the free parameters, the model was fit to the empirical data itself. Through the fitting procedure the values of the free parameters were obtained: they were the values that led to the best fit between the model and the data. This approach is valid, but there is a risk of overfitting and it is very computationally demanding. Here the causal inference model was tested with a new proposed approach. All model components were tested through numerical predictions and a generative model was avoided. We do so by proposing a new way of calculating the posteriors. This includes issuing predictions of distribution of estimates and of common causality.

The empirical study described in this manuscript analyzes and models the multisensory interactions in audiovisual distance perception. Psychophysical data were obtained in an experiment where visual and auditory stimuli of a person playing an organ were presented at several distances inside a room. The bimodal trials consisted of several stimuli distance combinations. Subjects reported both the perceived visual and auditory distance of the stimuli. The above mentioned causal inference model is tested against other multisensory models. Sensory weights are calculated according to a new formula that accounts for causal inference. This allows for the description of cue interactions at several cue positions and discrepancies in space.

## 1 Multisensory Modeling

### 1.1 Causal Inference

Causal inference in multisensory perception assumes that multisensory stimuli can stem either from the same source or from separate sources. Further detail on causal inference in multisensory perception can be found in an article by [18]. This approach can be decomposed into four problems: 1) calculating the percept if a single cause is assumed; 2) calculating the percept if separate causes are assumed; 3) finding out the probability of common and separate causes; and 4) calculating the final percept accounting for all previous steps. These four problems have been previously solved [20] using a generative model with several free parameters. Here we solve them in a similar way, but without a generative model. A *causal inference* model is proposed where predictions of variance are added and the multisensory estimates are modeled assuming normal distributions.

#### 1.1.1 Single Cause

When a single underlying event is inferred (*C* = 1), multisensory integration mechanisms can be predicted. We assume an unbiased perceiver whose estimates ${\hat{s}}_{i}$ can be well approximated by the underlying sensations *x*_*i*_. The underlying sensations can be indirectly observed in the unisensory estimates. Note that while spatial prior biases may exist in the formation of estimates of unisensory signals from external stimuli (e.g. [20, 21]), no such biases are known between the estimate and the underlying sensation, or from the sensation under unimodal stimulation to the sensation under bimodal stimulation. If any bias were to be observed in the bimodal condition, it is hypothesized that it would be due to the concurrent stimulus, and not due to a change in the prior of the sensation itself. We also assume the sensations *x*_*i*_ to have a normal distribution with parameters *N*(*μ*, *σ*) and that added sensory noise is independent across modalities. In this case, the estimates ${\hat{s}}_{i}$ can be calculated using the Maximum Likelihood Estimation (MLE) model [7, 19]:

$$\begin{array}{c} {\hat{s}}_{A,C=1}={\hat{s}}_{V,C=1}=\frac{\frac{x_{A}}{\sigma_{A}^{2}}+\frac{x_{V}}{\sigma_{V}^{2}}}{\frac{1}{\sigma_{A}^{2}}+\frac{1}{\sigma_{V}^{2}}} \end{array}$$ (1)

The benefit of multisensory integration is also observed in its predicted variance in which, when all assumptions are true, the inverse-variance-weighted estimate is also the minimum variance estimate of the stimulus property [7]:

$$\begin{array}{c} \sigma_{A,C=1}^{2}=\sigma_{V,C=1}^{2}=\frac{\sigma_{A}^{2}*\sigma_{V}^{2}}{\sigma_{A}^{2}+\sigma_{V}^{2}} \end{array}$$ (2)

#### 1.1.2 Separate Causes

When two causes are inferred (C = 2), sensory estimates are not affected by the concurrent sensory stimulation. Therefore, they can be approximated by the corresponding unimodal sensations:

$$\begin{array}{c} {\hat{s}}_{A,C=2}=x_{A}\;\text{and}\;{\hat{s}}_{V,C=2}=x_{V} \end{array}$$ (3)

In a similar way their variances correspond to the variance of the unimodal sensation. Therefore it is hypothesized that the variances of the estimates do not change from the unimodal to the bimodal condition, when two causes are inferred:

$$\begin{array}{c} \sigma_{A,C=2}^{2}=\sigma_{A}^{2}\;\text{and}\;\sigma_{V,C=2}^{2}=\sigma_{V}^{2} \end{array}$$ (4)

#### 1.1.3 Probability of Common Causes

In Eq (1) it is assumed that when there is a common cause sensory estimates are the same for both sensory modalities. Therefore, the causal probability can be indirectly observed by the similarity of the estimates ${\hat{s}}_{i}$. When the estimates are similar, a common cause can be inferred. A separate cause is inferred in the remaining cases:

$$\begin{array}{ccc} p(C=1|x_{V},x_{A}) & = & p(x_{A}=x_{V})\\ & and & \\ p(C=2|x_{V},x_{A}) & = & 1-p(x_{A}=x_{V}) \end{array}$$ (5)

#### 1.1.4 Calculating the Final Percept

The estimate of the probability of a common cause is rarely perfectly 0 or 1. When *p*(*C* = 1) is any number between 0 and 1, it must be determined how the estimates from Eqs (1) and (3) are combined. We test three strategies proposed by [20].

In the model selection strategy one may simply choose the estimate from the most likely causal structure:

$$\begin{array}{c} {\hat{s}}_{A|x_{V},x_{A}}=\{\begin{array}{cc} {\hat{s}}_{A,C=1} & \text{if}\;p(C=1)>0.5\\ {\hat{s}}_{A,C=2} & \text{if}\;p(C=1)<0.5 \end{array} \end{array}$$ (6)

Let us assume an example where a given stimulus pair has *p*(*C* = 1) = 0.3. According to model selection, in our example, the estimates would always follow the most likely causal structure, which is *C* = 2. The estimates would therefore follow a normal distribution with mean and variance as observed in the unimodal condition.

In the model averaging strategy subjects have access to both independent estimates and provide a combined estimate. The final estimate is a linear weighted average of the estimates from both causal structures.

$$\begin{array}{c} {\hat{s}}_{A|x_{V},x_{A}}=p\left(C=1|x_{V},x_{A}\right)*{\hat{s}}_{A,C=1}+p\left(C=2|x_{V},x_{A}\right)*{\hat{s}}_{A,C=2} \end{array}$$ (7)

In our example, the final auditory estimate would therefore be the sum of 30% of ${\hat{S}}_{A,C=1}$ with 70% of ${\hat{S}}_{A,C=2}$. To test this strategy, and in the absence of a better prediction from previous research, we also hypothesize that the variance of this estimate is a linear weighted average of the predicted variances for each causal structure.

$$\begin{array}{c} \sigma_{A|x_{V},x_{A}}^{2}=p\left(C=1|x_{V},x_{A}\right)*\sigma_{A,C=1}^{2}+p\left(C=2|x_{V},x_{A}\right)*\sigma_{A,C=2}^{2} \end{array}$$ (8)

A third strategy, probability matching, assumes that estimates vary from trial to trial, following either ${\hat{s}}_{A,C=1}$ or ${\hat{s}}_{A,C=2}$. Each estimate type occurs proportionally as many times as its causal probability:

$$\begin{array}{c} {\hat{s}}_{A}=\{\begin{array}{cc} {\hat{s}}_{A,C=1} & \text{if}\;p(C=1|x_{V},x_{A})>\zeta \\ {\hat{s}}_{A,C=2} & \text{if}\;p(C=1|x_{V},x_{A})<\zeta \end{array} \end{array}$$ (9)

where *ζ* is sampled randomly from the uniform distribution [0:1]. Returning to our example, in practice, the estimates will follow the mean and variance of *p*(*C* = 1) in 30% of the trials and of *p*(*C* = 2) in the remaining trials. It follows that the response distribution according to this strategy is composed of two gaussian distributions, each with the relative size of each causal probability.

### 1.2 Models without causal inference

All the strategies described above assume causal inference. Additional models that do not assume causal inference were also tested. This testing aimed at establishing if causal inference is likely to take place in the perception of visual and auditory distance. Three alternative explanatory models were tested. *Sensory dominance* was tested by assuming that the most reliable cue always takes over. Therefore, the sensory estimates ${\hat{s}}_{i}$ in the multisensory condition can be approximated by the perceived distance *x*_*i*_ in the unimodal condition that had the lowest variance, as given by

$$\begin{array}{c} {\hat{s}}_{A|x_{V},x_{A}}={\hat{s}}_{V|x_{V},x_{A}}=\{\begin{array}{cc} x_{A} & \text{if}\;\sigma_{V}^{2}>\sigma_{A}^{2}\\ x_{V} & \text{if}\;\sigma_{V}^{2}<\sigma_{A}^{2} \end{array} \end{array}$$ (10)

and in a similar manner the distribution of the estimates in the multisensory condition corresponds to the smallest distribution of the estimates of the unimodal sensation. *Mandatory integration* was tested by assuming that all estimates follow the linear inverse variance weighting rule. Therefore, the estimates in the multisensory condition are given by Eq (1), and their distribution is given by Eq (2). Finally, *no interaction* was also tested, where all estimates and distributions were given directly by the respective unimodal sensation, as described in Eqs (3) and (4).

### 1.3 Sensory Weights

Finally, we aimed at quantifying the relative contribution of each sensory cue by applying the principles of causal inference. In the conventional MLE model [7] it is assumed that the sensory weight is given by the normalized inverse of the variance of the unisensory estimate:

$$\begin{array}{c} w_{A}=\frac{1/\sigma_{A}^{2}}{1/\sigma_{A}^{2}+1/\sigma_{V}^{2}} \end{array}$$ (11)

To calculate the sensory weights accounting for causal inference one must calculate separately the weights for each causal structure. In the case of dual causality, it is assumed that the cues do not interact. Therefore, the weight of the auditory stimulus on the auditory distance estimate equals 1. In the case of a common causality, one may assume the weights as predicted by the MLE. Both in the probability matching and in the model averaging strategy, the average sensory weights correspond to the combination of the weights of perceived common (*p*(C = 1)) and separate (*p*(C = 2)) causes:

$$\begin{array}{c} w_{A}=w_{A,C=2}*p\left(C=2|x_{V},x_{A}\right)+w_{A,C=1}*p\left(C=1|x_{V},x_{A}\right) \end{array}$$ (12)

Therefore, the sensory weights can be calculated as follows:

$$\begin{array}{c} w_{A}=p\left(C=2|x_{V},x_{A}\right)+p\left(C=1|x_{V},x_{A}\right)*\frac{\sigma_{V}^{2}}{\sigma_{A}^{2}+\sigma_{V}^{2}} \end{array}$$ (13)

and

$$\begin{array}{c} w_{V}=p\left(C=1|x_{V},x_{A}\right)*\frac{\sigma_{A}^{2}}{\sigma_{A}^{2}+\sigma_{V}^{2}} \end{array}$$ (14)

where *w*_*i*_ is the weight of each sensory modality on a given auditory estimate.

## 2 Materials and Methods

### 2.1 Ethics Statement

**Fig 2:**
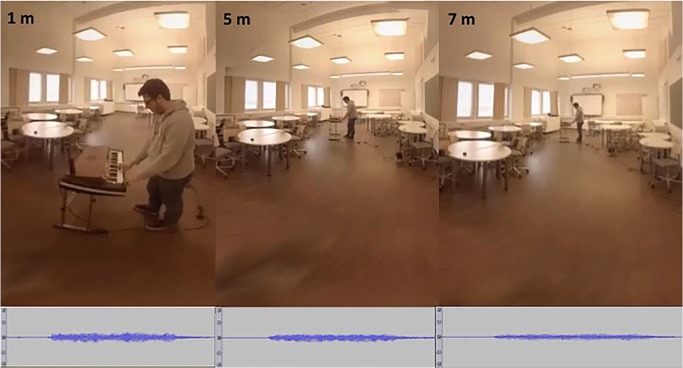
Stimuli at 1, 5 and 7 m in distance.

The experiment followed the policies on human subjects research as described in the Declaration of Helsinki. Participants provided written informed consent. The experimental protocol was approved by the Aalto University Ethics Committee. The individual in Fig 2 of this manuscript is an author and has given written informed consent (as outlined in PLOS consent form) to publish this figure.

### 2.2 Participants

There were six participants. One participant was a female and one participant was one of the authors. Participants had normal hearing and vision. Participant age ranged from 21 to 33.

### 2.3 Stimuli

There were three blocks of experiments, each corresponding either to the audiovisual, the visual, or auditory experimental conditions. All subjects performed the audiovisual block first. The auditory and visual conditions were tested in one single session two months later. The visual and auditory blocks were counterbalanced in order across participants. The rationale behind this method was that all participants did the audiovisual localization trials—the main condition under study—without any prior training or knowledge of the stimuli. The two-month period between the sessions intended to create a gap in which the subjects would forget any learning occurred in the first session. Therefore, all conditions were run without interference of knowledge from other conditions.

Visual and auditory stimuli consisted of immersive reproductions of a young man playing an electronic portable organ in a room. In the visual condition only video, and no sound, was presented. In the auditory condition only sound was presented. In the audiovisual condition both video and sound were presented. Both visual and auditory stimuli were recorded in a large classroom, 10.9 m long by 6.5 m wide, and 2.2 m high. The reverberation time of the room was 1.9 sec. Stimuli were recorded along the 12.7 m room diagonal. Both the cameras and the microphone were positioned in one corner of the room, 1 m away from each wall, and at a height of 1.6 m. Recordings were taken of a male playing the C chord on an organ at different positions along the room diagonal.

The visual stimuli were recorded with 6 GoPro cameras mounted on a cubical support Freedom 360. With Autopano Video software the 6 videos were stitched together and thus a spherical 360 deg video was obtained. Visual stimuli were reproduced with an Oculus Rift device and allowed for free head movement with realtime rendering of the room. Auditory stimuli were recorded with an Eigenmike microphone (32 channels) reduced to first-order B-format (4 channels) rotating the axis in order to match video and audio directions. The Eigenmike microphone was chosen due to its structure, which has higher aliasing frequency than other B-format microphones available. Recording was rendered to create a 3D real-time audio environment using Directional Audio Coding [22, 23]. The auditory stimuli were reproduced through a set of Sennheiser HD 650 headphones. The audio was synchronized with the video using a cross correlation function. Recordings lasted for 3.3 sec and the organ was played continuously for 2 sec, starting at sec 0.7. The reason for the long stimulus duration had to do with the need to include enough information for subjects to access the direct-to-reverberant energy ratio and to access the full room reverberation. Both cues are known to be critical in auditory distance perception. There was a metronome playing in the background, which was positioned at the same distance as the organ. Its purpose was to time the keypress in all recordings. The visual stimuli consisted of random presentations of the spherical recording of the organ being played at 1, 3, 5, 7 and 9 m from the camera (Fig 2). The auditory stimuli consisted of random presentations of the sound environment of the organ being played at every meter, from 1 to 10 m in distance from the microphone. The audiovisual stimuli consisted of all the possible combinations of visual and auditory stimuli, randomly presented. In all conditions there were six repetitions per stimulus.

### 2.4 Apparatus and Procedure

Experiments took place in an acoustically treated room. Participants were seated on a chair, with the Oculus Rift placed over the eyes and the headphones over the Oculus Rift (Fig 2). The image was centered so that the stimuli were presented straight ahead. The visual, auditory, and audiovisual conditions were tested in separate sessions. The audiovisual condition was tested in two sessions. Before the beginning of each condition participants had a practice block. In the practice block, each stimulus was presented once in random order. Participants were instructed to pay attention to the room and to the stimuli, and to provide responses in the response interface. The response interface consisted of an iPad containing two sliders and one Continue button. One slider allowed to input responses to the visual stimuli, and the other one to the auditory stimuli. There was only one slider in the visual and auditory condition. The iPad interface and input could be seen on the Oculus Rift. By moving the slider participants could choose any value, ranging from 0 to 10. Participants were told that 0 corresponded to their own position in space, that 10 corresponded to a position just before the corner at the other end of the room, and that the values corresponded to actual meters in the room. Participants were asked to always answer to both visual and auditory distance after each audiovisual trial, but they were allowed to choose which one to respond to first. They were specifically asked to always provide the same answer if both stimuli were perceived as stemming from the same point in space. This was important, as the rate of matching answers was used to compute the probability of perceived common cause in the causal inference model. Participants were further asked to provide the most honest report of what they actually perceived, not what they believed was the correct answer. This instruction had to do with the fact that there was no time limit to provide answers. It aimed specifically at asking subjects to avoid developing theories of what the experimental design might be and to avoid trying to find the correct answer, instead focusing in simply reporting percepts. Each trial started after pressing the Continue button.

### 2.5 Statistical Tests

To analyze the effect of each sensory modality over the visual and auditory distance estimates, a Kruskal-Wallis test was used. The choice of a non-parametric test had to do with the small number of subjects (n = 6). To test the multisensory models, a simple linear regression test was used. All pooled data were used, organized by subject, corresponding to the response counts per each of the 10 points in space in each of the 50 stimulus pairs per subject, fit to the corresponding predicted response counts (10 distances * 50 stimuli * 6 subjects = 3000 data points). Residuals were found to be normally distributed. Linear regressions were used because data were linearly related to the models. No model correction, such as Akaike’s criterion, was used because all models had the same number of parameters in the fitting procedure and no generative model was used. Separate linear regressions were calculated for each model, for the visual estimates/predictions, the auditory estimates/predictions, and both. Separate regressions were also calculated for each subject.

## 3 Results

Overall, the localization of visual stimuli in distance was more accurate than that of the auditory stimuli. In the unimodal visual condition the average localization error was 0.34 m (0.49 SD), while in the unimodal auditory trials, the average localization error was 1.42 m (1.28 SD). In audiovisual trials, visual distance estimates had an average error of 0.33 m (0.51 SD). The auditory distance estimates in that condition had an average error of 1.48 m (1.33 SD). In bimodal trials, the perceived auditory distance was significantly affected both by auditory stimulus distance (*χ*^2^_(9,5)_ = 1040.01, *p* = 0.000) and visual stimulus distance (*χ*^2^_(9,5)_ = 20.52, *p* = 0.000). However, the perceived visual distance was only affected by visual stimulus distance (*χ*^2^_(9,5)_ = 1731.23, *p* = 0.000) and not by the auditory stimulus (*χ*^2^_(9,5)_ = 0.28, *p* = 1.000).

Multisensory integration was modeled using *causal inference*. For each stimulus pair, we: 1) calculated the mean (Eq (1)) and variance (Eq (2)) of the percept for a single underlying cause; 2) calculated the mean (Eq (3)) and variance (Eq (4)) of the percept for two underlying causes; 3) quantified the probability of common and separate causes (Eq (5)); and 4) calculated the final percept accounting for all previous steps (Eqs (6)–(9)). In the calculation of the final percept three strategies were tested: probability matching (Eq (6)), model averaging (Eqs (7) and (8)) and model selection (Eq (9)). Three additional models were tested: *sensory dominance* (Eq (10)), *mandatory integration* (Eqs (1) and (2)) and *no interaction*.

**Fig 3:**
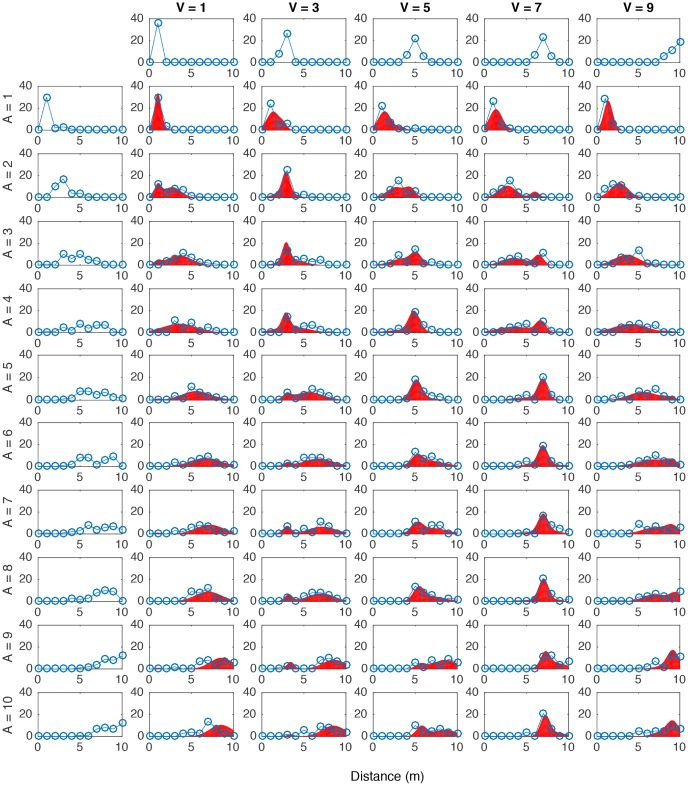
Auditory distance response distributions in all bimodal conditions (blue connected circles) and response distributions as predicted by *Causal Inference* resolved with the probability matching strategy (red area). The topmost graphs correspond to the unimodal visual distance distributions. The leftmost graphs correspond to the unimodal auditory distance distributions. Distributions were obtained by pooling all responses from all subjects. Auditory stimulus distance ranges in rows from 1 m distance (A = 1) to 10 m (A = 10) and visual stimulus distance ranging in columns from 1m to 9 m (V1 to V9 respectively).

A visualization of the multisensory mechanisms simulated in each model is presented in Fig 1. The predicted distributions for each model were generated and tested against the actual response distributions. In Fig 3 all of the obtained auditory distance responses ${\hat{s}}_{A}$ in each multisensory trial type are presented together with the predictions from the probability matching strategy. The visual and auditory unimodal pooled distributions are also presented at the topmost and leftmost graphs, respectively. It is observed that the probability matching strategy constitutes a close approximation to the average auditory distance responses.

**Fig 4:**
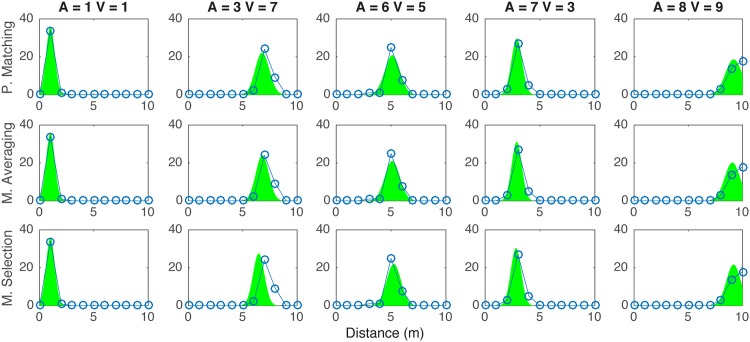
Five examples of visual distance response distributions (blue connected circles) and corresponding response distributions as predicted by *Causal Inference* resolved with the probability matching, model averaging, and model selection strategy (green area). Response distributions obtained from all pooled data.

**Fig 5:**
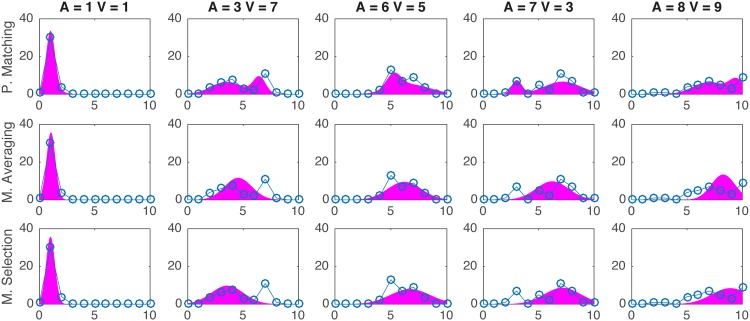
Five examples of auditory distance response distributions (blue connected circles) and corresponding response distributions as predicted by *Causal Inference* resolved with the probability matching, model averaging, and model selection strategy (magenta area). Response distributions obtained from all pooled data.

Figs 4 and 5 present visual and auditory distance responses in a sample of trial types against the predictions from the probability matching, model averaging and model selection strategies. In Table 1 a summary of all model fitting is presented. Looking at all data together (All), the *causal inference* models explained the data better, followed very closely by the *no interaction model*. The probability matching strategy yielded the best fits. This was also true in the data from each individual subject: for all subjects, the best fits were obtained with the *causal inference* model resolved with probability matching. In general, better fits were obtained for the visual distance estimates than for the auditory estimates. This may be related to the fact that visual estimates were more consistent and had lower variance that auditory estimates. The visual estimates were well predicted by all tested models, and the best fit values was obtained with the *causal inference* model resolved with model averaging.

**Table 1:** Goodness of fit of each model (*r*^2^).

Model	All	All (A)	All (V)	s1	s2	s3	s4	s5	s6
*SensoryDominance*	0.451	0.121	0.853	0.435	0.462	0.66	0.530	0.411	0.447
*MandatoryIntegration*	0.448	0.074	0.952	0.378	0.475	0.469	0.506	0.410	0.472
*NoInteraction*	0.838	0.613	0.838	0.934	0.874	0.780	0.775	0.755	0.851
*CausalInferencePM*	**0.863**	**0.679**	0.963	**0.938**	**0.885**	**0.804**	**0.826**	**0.792**	**0.883**
*CausalInferenceMA*	0.841	0.618	**0.964**	0.935	0.869	0.778	0.790	0.743	0.872
*CausalInferenceMS*	0.849	0.642	0.963	0.935	0.872	0.787	0.795	0.790	0.867

Goodness of fit of each model against all distance estimates (All), the auditory distance estimates (All (A)) and the visual distance estimates (All (V)). Causal inference was tested with three strategies: probability matching (PM), model averaging (MA), and model selection (MS). The goodness of fit was obtained by simple linear regression with ordinary least squares regression. The regression analysis fit predictions against observed response rates per stimulus type and subject.

**Fig 6:**
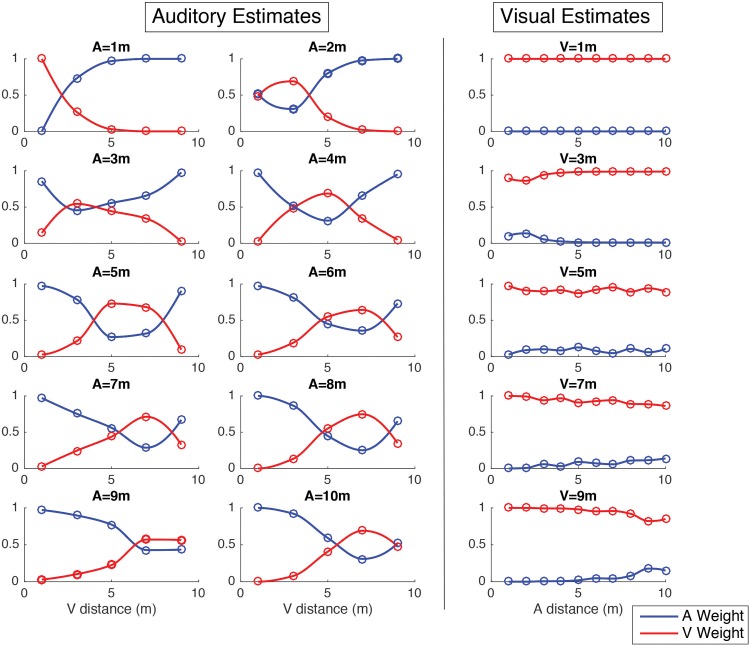
Sensory weights in auditory distance estimates. Calculated from all pooled data.

In a final step, sensory weights were calculated accounting for causal inference. The sensory weights were calculated as described in section 2B (Eqs (13) and (14)). They were calculated for each stimulus pair by averaging across all pooled data. The calculation followed the steps: 1) calculating each sensory weight for a single underlying cause; 2) calculating each sensory weight for two underlying causes; and 3) calculating the average of each weight accounting for the probability of each causal structure. In Fig 6 the sensory weights for the auditory distance estimates are presented for each stimuli pair. It can be observed that at all distances there is a window within which the visual cue largely affected the auditory estimate. This window of interaction is mobile and centered around the auditory stimulus position, meaning that when both stimuli were in close proximity they interacted in the formation of the auditory estimate. In those cases the auditory estimate was pulled in the direction of the visual stimulus. We can quantify the window size as the area where the weight of the visual cue surpasses that of the auditory cue. We find that the smallest window is observed with the auditory stimulus at 1 m, and it is 1 m wide. The largest interaction window occurs when the auditory stimulus is at 5 m, and is 4 m wide. The average interaction window is approximately 3 m, and stimuli interact little outside of it. The sensory weights in the visual distance estimates were markedly different. At all distances, the visual cue was the most weighted. The highest visual weights were found when the visual stimulus was at 1 m, where they were always equal to 1. The lowest weights were observed with the visual stimulus at 9 m, where the visual weight was close to 0.8 when the auditory stimulus was at 9 and 10 m.

## 4 Discussion

### 4.1 Audiovisual Distance

We studied the perception of distance from visual and auditory stimulation. It is remarkable that so little attention has been paid to multisensory interactions taking place in the extrapersonal space [24]. Little is known about the cue interactions in the audiovisual perception of distance. It is known that for visual and auditory events to be perceived as synchronized the auditory stimulus must lag the visual stimulus accounting for sound propagation velocity [25–27]. Previous research shows some potential audiovisual interactions. A cueing sound at the same distance as a visual target enhanced the detection of the visual target [28]. It has also been reported that having visual references about the space improves auditory distance localization [29]. Seeing all response options, namely seeing an array of loudspeakers, improves localization of sounds in distance [30], while seeing only one loudspeaker biases the perceived distance toward it [31–34]. In those studies there were never visual events to relate the auditory events to. Therefore, the localization of visual and auditory events in distance when presented together had never been analyzed. The impact of sound events on visual distance perception remained unexplored, too, and it was not known how visual and auditory cues interact under congruent and incongruent conditions, and at several distances. In our experiment, subjects were immersed in an audiovisual recorded room and were allowed to move their heads, since stimuli were generated in realtime. Therefore all sensory distance cues were available, except for binocular rivalry.

We found that visual distance estimates were for the most part accurate and that the impact of auditory cues was negligible. Auditory information had a maximum weight of 20 percent over the visual distance estimate, namely when stimuli were presented in proximity of each other and at large distances. In the bimodal trials it was found that vision has a large impact over the perceived auditory distance, contributing with more that 50 percent of the weight, namely when stimuli are presented within 3 m of each other. With larger stimuli separations the perceived auditory distance is mostly unaffected by visual stimulation. In light of the *causal inference* model, this window of interaction can be interpreted as the window within which humans alternate between inferring one and two separate external causes.

The fact that auditory cues had such low impact on visual distance estimates, and that visual cues only affected the auditory estimates within well defined windows may be related to the choice of stimuli employed in this experiment. In fact, stimuli were relatively longer in duration when compared to other audiovisual localization experiments. This choice of duration had to do with the fact that two main cues for auditory distance localization are reverberation time and direct-to-reverberant energy ratio [35–39], and reverberation as cue has a longer duration. Other auditory distance cues include sound level, auditory parallax, high frequency components and high room reflectance [35, 37, 40–43]. Our study included all of the above cues. This may certainly have increased the accuracy of the auditory image and therefore reduced uncertainty, which in turn may have contributed to lowered cue integration, higher rates of perceived separate causes, and generally low levels of cue interaction. In a similar way, our visual stimuli were very rich in distance cues: retinal size, familiar size, parallax, optic flow, texture gradient, and light and shade were available (see [44] for an overview on visual distance cues) and stimuli duration was enough for all cues to be processed with accuracy. Therefore, perhaps if stimuli duration was shorter, or if stimuli were impoverished and abstract, higher perceived common causality could have been obtained, or higher interaction observed. On the other hand, the use of realistic stimuli under well-controlled conditions can provide an insight on the mechanisms of multisensory combination as they often occur. It also provides for a preservation of stimulus identity from a common audiovisual source, which promotes multisensory binding [45]. From this point of view it is equally arguable that realistic and congruent stimuli could be associated to higher levels of perceived common causality and integration than if more abstract stimuli were used.

### 4.2 Multisensory Models

To assess the multisensory interactions in distance perception we tested several multisensory models. One of these models, the *causal inference* model, was tested with a different approach from previous multisensory studies. The model predicted that when separate causes are inferred the estimates have the same distribution as in the unisensory condition. When common causes are inferred it predicted that the distribution of the estimates can be given by the MLE model. It also proposed possible distributions of the combined estimates. While other distributions may be equally or more valid, we found that all tested *causal inference* models fit the data well. Three other longstanding models that do not assume causal inference were tested. It was found that overall *causal inference* fit better to the data, closely followed by the *no interaction* model. *Sensory dominance*, the longest standing model, explained the smallest portion of the overall data. *Mandatory integration*, which is still widely used as the main current model in multisensory processing, was the second worst. Note that mandatory integration is also a part of the causal inference model. In the *causal inference* model it would be expected to occur if a common source was often inferred. Therefore, it is likely that the poor performance of the *mandatory integration* model in explaining the results from our experiment has to do with the low rates of perceived common causality. The *no interaction* model explained more than 80% of the overall data and did considerably well across all subjects. This model, too, is partly integrated in the *causal inference* model. It is expected to take place when two causes are inferred. Since in the experiment we report there were high rates of perceived separate causes, the good performance of the *no interaction* model is not surprising. The *causal inference* yielded the best fits in every tested case. Resolved with the probability matching strategy, it was found to explain the largest proportion of the overall data, which is in line with the finding of the study that proposed these resolving strategies [20]. In fact, when observing the response distributions of the auditory distance estimates it is often observed that there are two peaks. This bimodal distribution corresponds well to the estimates of each causal structure and it is a key feature in the *probability matching* strategy. It can be therefore hypothesized that, instead of combining each of the estimates from the inferred causal structures, perceivers alternate their response strategy between one and the other. All subjects seemed to follow more the probability matching strategy, which is not to say they did not alternate between strategies during the experiment. Indeed, looking at the visual distance estimates separately, we see that model averaging did slightly better, although all models fit very similarly. [20] compared the three causal inference strategies in a large population of subjects for localization of audiovisual horizontal stimuli. They found that the majority of the subjects followed more the probability matching strategy, while others followed more a model averaging or model selection strategy. A model averaging strategy means that subjects respond mostly according to a linear weighted average on the two causal structures, while a model selection strategy means that subjects respond mostly according to the most probable causal structure. It must be noted that here these strategies were tested with different calculations from the study by [20]: no generative model was used, and instead model predictions were obtained by using the same calculations for response centroid, but original calculations for response distribution. Therefore result comparisons between studies should be read with caution. The models used here seem however to work as a plausible alternative to test the *causal inference* model with much reduced computational complexity and demand.

In a final step, with the purpose of quantifying the overall importance of each sensory cue over the other, we proposed a new method to calculate sensory weights. This computation allows for an intuitive visualization of the interactions between cues in all tested cue combination cases. It also allows for the quantification of the relevance of each cue, and for the measurement of the window of interaction. Here, the window of interaction was defined as the stimuli range within which the sensory estimates of one sensory modality were affected in more than 50% by another concurrent sensory modality. In this case, it was observed that there is a clear window of interaction in auditory distance perception, which is always centered around the stimulus position itself. However, there is no such window in the perception of visual distance.

Taking an overview of our data, they seem to suggest that visual distance and auditory distance percepts are formulated though different mechanisms. The sensory weights of each cue are different for visual and for auditory distance estimates, even when they are mostly perceived as co-localized. Also, the multisensory model that best explains estimates in one sensory modality might not be best suited for the other sensory modality. This possibility calls for the need of analysing the multisensory percepts in each modality separately, and for the testing of multisensory models with this in mind.

Several notes should be taken while reading our test of multisensory models in the perception of audiovisual distance. Firstly, it must be noted that the performance of each tested model against our data may have been influenced by the research method itself. It may be that the fact that only 10% of the trials presented co-localized stimuli decreased the probability of inferred common causality. It may also be that the availability of very clear distance cues combined with a long stimulus duration might have decreased the level of cue interaction. However, it is also true that currently all research testing the *causal inference* model uses similarly low rates of congruent stimuli. Indeed, even articles testing other multisensory mechanisms such as the MLE tend to exhibit this limitation. In any case, it remains an open question whether the rate of congruent stimuli would affect model performance, and future studies should address this issue. It may also be that the availability of most distance cues, combined with long stimulus duration, may have affected the level of cue interaction. This is also an open question in most research experiments in this field, and only a new battery of tests manipulating stimulus quality would be able to answer it. Nevertheless, the *causal inference* model is sensitive to changes in rate of cue integration, by simply assuming different values of probability of common cause, and it can therefore be expected that the model would perform equally as well with different stimuli arrangements and experimental designs.

Finally, the proposed *causal inference* model was merely a first suggestion of how perceptual causal inference mechanisms might be estimated with simple mathematical formulations. There is the need to further look into this model and explore the existence of potential biases, test alternative distributions, and alternative resolution strategies. The proposed model should also be tested against other *causal inference* model formulations and other datasets.

## 5 Conclusion

The models of multisensory integration are a very useful tool to describe quantitatively how different sensory cues interact to produce a sensory estimate. Here, these models are brought forward to explain the data from an experiment on the audiovisual perception of distance. The *causal inference* model with probability matching strategy approximated the overall data better than the other models. The causal inference principles can also be used to calculate the sensory weights under all stimuli combinations. These weights revealed that, within a given window, the visual cue has greater importance than the auditory cue in the perception of auditory distance. The visual cue is the most prominent one in the perception of visual distance.
